# Community and facility-level engagement in planning and budgeting for the government health sector – A district perspective from Kenya

**DOI:** 10.1016/j.healthpol.2010.08.027

**Published:** 2011-03

**Authors:** Wendy Prudhomme O’Meara, Benjamin Tsofa, Sassy Molyneux, Catherine Goodman, F. Ellis McKenzie

**Affiliations:** a Duke University School of Medicine and Duke Global Health Institute, Durham, NC, USA; b KEMRI-Wellcome Trust Research Programme, Kilifi, Kenya; c KEMRI-Wellcome Trust Research Programme, Nairobi, Kenya and Health Policy Unit, London School of Hygiene & Tropical Medicine, London, UK; d Fogarty International Center, National Institutes of Health, Bethesda, MD, USA

**Keywords:** Health planning, Decentralization, Community participation

## Abstract

Health systems reform processes have increasingly recognized the essential contribution of communities to the success of health programs and development activities in general. Here we examine the experience from Kilifi district in Kenya of implementing annual health sector planning guidelines that included community participation in problem identification, priority setting, and planning. We describe challenges in the implementation of national planning guidelines, how these were met, and how they influenced final plans and budgets.

The broad-based community engagement envisaged in the guidelines did not take place due to the delay in roll out of the Ministry of Health-trained community health workers. Instead, community engagement was conducted through facility management committees, though in a minority of facilities, even such committees were not involved. Some overlap was found in the priorities highlighted by facility staff, committee members and national indicators, but there were also many additional issues raised by committee members and not by other groups. The engagement of the community through committees influenced target and priority setting, but the emphasis on national health indicators left many local priorities unaddressed by the final work plans. Moreover, it appears that the final impact on budgets allocated at district and facility level was limited. The experience in Kilifi highlights the feasibility of engaging the community in the health planning process, and the challenges of ensuring that this engagement feeds into consolidated plans and future implementation.

## 1 Background

The Alma Ata declaration on primary health care [1] stated that “people have the right and duty to participate individually and collectively in the planning and implementation of their health care”. This has been one of the factors leading to increased emphasis on decentralization in health care, which has become a common theme in health system reform. Decentralization can be defined as allocating more decision-making responsibilities from the national level to the peripheral levels. Within the health sector, this typically means bringing the decision making closer to the implementation level. Community participation in the health sector is often seen as an integral part of decentralization and essential for achieving high coverage and equitable distribution of health services. Community participation has re-emerged as a priority in health service delivery in an effort to move ‘away from [considering] users as recipients of services designed for their benefit, towards communities being active makers and shapers of services, exercising their preferences as consumers and their rights as citizens’ [2]. Strengthening community accountability in health systems is promoted as a right in itself, and for its potential to enhance quality of care, appropriateness of health service delivery for users, and ultimately patient satisfaction and utilization [2], [3], [4], [5]. On the 30th anniversary of the Declaration of Alma Ata, the WHO renewed its call for health for all through comprehensive primary health care [6]. In light of this, effective community participation will likely remain at the forefront of the health reform agenda.

Rifkin [7] defines five levels of community participation, ranging from very narrow participation where communities act as recipients of health services, to increasingly active roles with more responsibility including community involvement in implementation, evaluation and monitoring. The highest level of participation is described as community involvement in planning. Despite the prominence of decentralization and community accountability in policy, there is little published experience, leading to calls for further research [2], [5].

The Government of Kenya has been decentralizing its health sector to the district level since the early 1980s. This has included building capacity at the level of district administration so that planning, budgeting, and monitoring activities are transferred from the national level to the District Health Management Team (DHMT) [8]. Over the last four years, planning and budgeting at the district level has been introduced in a step-wise process. During the planning process for fiscal year 2005–2006, districts were asked to develop budgets and work plans for health activities according to a specific set of guidelines. In subsequent years, the district-level planning process evolved to become more comprehensive. During the 2007–2008 annual planning cycle, districts were asked to develop joint work plans in partnership with other entities (i.e. NGOs and private organizations) operating within the health sector. In the 2008–2009 planning cycle, the MOH further devolved decision making to the facility level by requiring that planning and budgeting be done at each individual facility with active involvement of the community. The goal of this approach is to ensure that planned activities reflect priorities and capacity at the implementation level. Kenya is therefore aiming to make relatively radical strides in ensure decentralization and community participation in health systems.

Here we describe the national guidelines for the 2008–2009 annual planning process and discuss how they were implemented in Kilifi district in Kenya's Coast Province. We highlight strengths and weaknesses of the planning process as it relates to capacity at the implementation level and the engagement of communities in setting priorities within the health sector.

## 2 Methods

Kilifi district is located in the Coast Province of Kenya and is a typical rural Kenyan district with one small urban center. The DHMT manages 25 primary care facilities (dispensaries and health centres) and one referral hospital that serve a population of 600,000 people in the district. Here we offer reflections on the Ministry of Health 2008–2009 annual planning and budgeting process. Observations about the process were contributed by members of the DHMT and participants at the district level through meetings and group discussions. Observations collected in these forums were recorded by one of the authors (WPO) who was present during each stage of the process from the initial training of the DHMT to the final work plan and budget consolidation. WPO provided input into the translation of the planning guidelines for implementation in the district, but was otherwise not actively involved in the planning process.

In addition to observations collected during the process, there were three other sources of information. First, a document review of national guidelines for the planning and budgeting process. Second, an in-depth review of all minutes produced during meetings of facility committees related to the planning process. Finally, data on baselines and targets were extracted from the work plans produced by each health facility during the planning process. Baseline data were cross-checked with the Health Management Information System managed by the District Health Management Team.

## 3 National guidelines for planning and budgeting

**Fig. 1:**
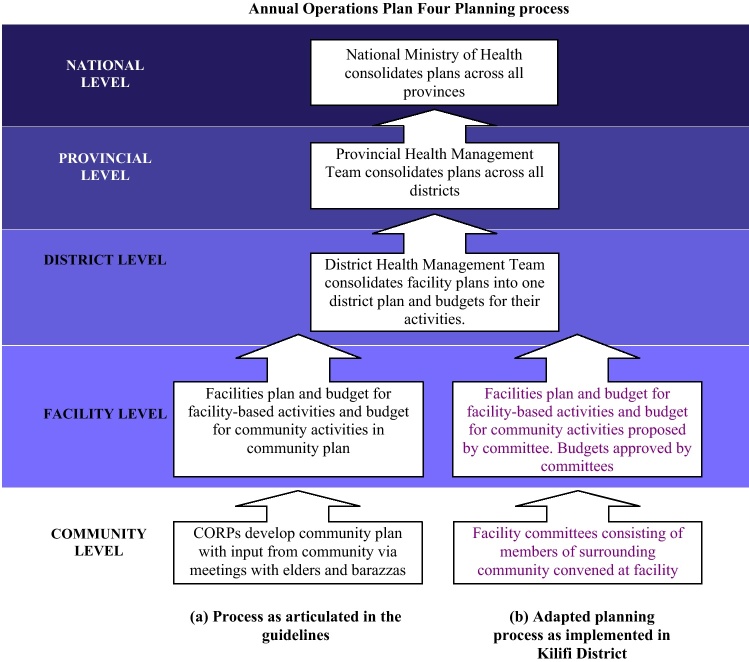
Inputs to the health sector working plan as outlined in the operational guidelines (a), and as implemented at the district level (b).

National guidelines laid out the objectives and methodology for the 2008–2009 annual planning process (also called Annual Operations Plan Four or AOP4). The planning process was to begin at the community level with the development of the community plan, coordinated by the community health workers (called Community Owned Resource Persons or CORPs) (Fig. 1). According to the MoH 2005 Community-level strategy [9], one CORP should be recruited per 50 households. The CORP and the 50 households are called the “Community Unit”. CORPs are overseen by Community Health Extension Workers (CHEWs) who operate from the primary care facilities (dispensaries and health centres). The first step in the planning process was for the CORPs to meet with the community elders to describe the health situation in the community, including major causes of morbidity and mortality, quality and availability of health services, and actions being taken by the community to improve health. This information was to be presented during a community dialogue day when community members, local stakeholders, and community health workers would develop a plan to address these issues and prioritize activities. It would then be the responsibility of the CORPs and CHEWs to synthesize a detailed list of activities and targets based on the feedback from the community dialogue day. This list of prioritized activities and targets for the upcoming year would be the community-level plan and would be incorporated into the facility plan by the facility in-charge.

Once the community-level plan had been generated, the facility would begin its planning process, starting with a situation analysis in which major constraints and challenges to service provision would be listed, followed by an evaluation of baseline performance across standardized service delivery indicators from the preceding year and setting targets for the upcoming year across the same indicators. The formats provided in the guidelines for entering targets were standardized and only included national indicators. Finally, each facility would generate a list of activities to meet their targets and develop a budget for each activity. The facility plan would incorporate the community-level plan and 25% of the facility budget would be earmarked for community activities as outlined in the community plan. Overall budgets for each facility were restricted by an expected allocation or “resource envelope” that was decided in advance based on the type of facility and the size of the population served by the facility.

The role of the district health management team was to train and assist facilities to carry out the planning process and to consolidate plans from all the facilities into a single district plan.

The AOP4 planning process was intended to lay the foundation for the national roll out of direct facility funding (DFF) for each health centre and dispensary, with Facility Management Committees responsible for planning the use of such funds. This system of DFF was already being piloted in Coast Province, but was expected to be rolled out nationwide in 2007–2008.

## 4 Implementation of the guidelines in Kilifi District

Observations made during meetings of the DHMT and discussions with key personnel revealed that the DHMT faced several challenges in implementing the guidelines. First and foremost was the absence of any trained CORPs in the district. Lack of funding had slowed the process nationwide and the MoH Community Strategy had not been launched. The DHMT decided to solicit the input of community representatives through the facility committees which had been established previously at every health facility. These committees are comprised of the health facility in-charge and one representative from each village surrounding the facility. The facility in-charge was asked to call a meeting of the facility committee to discuss the facility's annual plan. The standardized planning formats asked facility staff and community members to generate a list of barriers to accessing health and health services and to distinguish between barriers on the supply side versus the demand side. These barriers were discussed for five “life stages” – pregnancy and newborn, early childhood, late childhood, adolescence, and adulthood. A list of questions was generated by the DHMT to help guide the committees’ discussions (Box 1) in place of the barazaas (public meetings) recommended by the guidelines.

Box 1 **Questions to guide discussion with the facility committee.**

**Table:** 

1. What kinds of illnesses or health problems are most common among children?
2. Are the causes of death different than the causes of illness?
3. What kinds of illnesses are most common among adults?
4. Is it common in your community for mothers to go to ANC clinic? Early or late in pregnancy? Why?
5. Is it common for women to give birth in a facility? Why?
6. Do you think most people in your community know their HIV status? Do most people want to know? Why or why not?
7. What kinds of health concerns do the elderly have?
8. Do adolescents feel confident or welcome to access health services? Why or why not?

The second major challenge was the time constraints of the planning process. Planning was initiated late due to government reorganization following the contested elections in December 2007. National-level policy makers met and developed the guidelines in March 2008 and then trained Provincial Health Management Teams (PHMT). The PHMT of Coast Province offered a three-day training to all the DHMTs in the province. The teams were given four weeks to return their consolidated district work plans. The DHMT simplified and revised the guidelines to match the implementation context (i.e. using facility committees rather than community units and CORPs). One week later, the facility in-charges were called for a meeting during which they were trained on the revised planning process. They were asked to convene their committees and return their plans and budgets within one week. Following the meeting, DHMT members traveled to most of the facilities to help guide and facilitate the planning process. 15 of 22 facilities convened their committees and all of these produced minutes of the meeting during which they discussed barriers to accessing health care and the annual operations plan. The remaining facilities were not able to convene their committees for the planning process within the required timeframe, but still produced workplans based on input from facility staff. The facilities’ plans were submitted and the DHMT had to review the plans and compile them within one week. This did not allow any reiterative steps within the planning process, feedback to individual facilities, or capacity building in budgeting and planning.

## 5 Community participation in identifying priority health issues

The community perspective was brought by the facility committee, in which each village is intended to be represented by a committee member. Meeting minutes were reviewed and observations were solicited from the DHMT staff who attended the meetings. The minutes indicated that the committees discussed barriers to health at each life stage, using the questions developed by the DHMT and the tables from the guidelines issued by the MoH. Key health issues and barriers identified by community members were diverse and revealed an integrated view of factors that contribute to healthy communities. They discussed concerns ranging from health education, to environmental determinants of health, to water and sanitation, to mental health and health-seeking behavior (Box 2). Many of the themes were repeated; every committee identified health education needs, 8 out of 15 were concerned about drug and alcohol abuse, malaria and skin infections were often cited as priority problems for children, and malnutrition was a concern for both children and the elderly. Problems facing adults were divided evenly between chronic and infectious diseases.

Box 2 **Community priorities (based on minutes from facility management committee meetings at the 15 facilities where these took place).**

• All committees reported **health education** needs in their community
• Half identified **sanitation, clear water and environmental** constraints to good health and oriented solutions towards these things (such as digging latrines, demonstrations on water safety). One committee pledged to set an example by digging latrines at their own homes
• The majority of committees identified **low or late ANC attendance (*****n*** **=** **11) and home deliveries (*****n*** **=** **12)**, respectively, as a health concern in their community. Reasons included lack of space and equipment at the facility (*n* = 10), fear of HIV testing (*n* = 7), male midwives (*n* = 5), and distance from the facility (*n* = 13)
• Ten said that **malaria** was still a problem for children, but almost as many said that **malnutrition** was a serious problem
• Distance to a facility (*n* = 8), staff shortages (*n* = 8) and stock-outs (*n* = 6) were the major constraints to children being treated effectively for illnesses
• **Hydrocel/elephantitis** was identified as a problem by 3 committees
• Eight community committees cited **drug and alcohol abuse** as a problem for either adults or adolescents
• Some committees were concerned about **nutrition amongst the elderly** (*n* = 3) and that they had no one to look after them and suffered from neglect (*n* = 5)
• Problems facing adults are balanced between **infectious** diseases (TB, diarrhea, HIV) and **chronic** conditions (hypertension, diabetes)
• Communities suggested approaches to increase service utilization amongst adolescents including **school-based VCT** and **youth representation on facility committees**

After meeting with the facility committee, facility staff was required to give their own views on major health issues and the barriers to delivering health services on both the demand and supply side. These views were recorded in the facility's workplan. Both the minutes of the facility committee meeting and the workplans were reviewed and compared. Generally, minutes indicated that facility staff focused more narrowly on health service delivery and health concerns that could be dealt with at the facility. In 11 out of 22 facilities health workers described specific training needs for their staff, and most listed basic infrastructure problems. They also reiterated some of the concerns that communities raised such as need for health education, low ANC attendance, and malaria treatment and prevention. However, there were some notable differences. In almost every facility health workers prioritized childhood immunization and identified problems such as stock-outs and incomplete immunization, although committees rarely mentioned immunization. Both facility staff and community members were concerned about the number of pregnant women delivering at home. However, health workers listed barriers on the community side (such as education and cultural beliefs) whereas the community members focused on facility barriers such as lack of adequate space and staff and male nurses. Malnutrition was discussed by more than half of the committees, but was not mentioned by any facility staff.

## 6 Impact of committee participation on target setting and prioritization

After reviewing the issues raised by the committees and the staff, facility staff then set targets for specific service delivery indicators for the coming year. The indicators were provided in the national planning guidelines (Table 1). Coverage levels for most indicators were available for the preceding six months for each facility from routine Health Management Information Systems (HMIS); these were taken as the ‘baseline’. Facility workplans showed that the targets for the coming year were set for each indicator relative to the baseline, either equal to or exceeding current levels, although in a very few instances targets were set below baseline.

**Table 1:** Service delivery indicators for each life stage and percent increase set by facility workplans in Kilifi district.

	District wide targets				Facility targets^a^			
	Eligible population	Baseline	Target	Coverage at baseline	Coverage at target	Percent change from baseline to target	Maximum change in coverage	Minimum change in coverage
Maternal health								
Number of pregnant women receiving two doses intermittent preventive therapy for malaria (IPTp)	15,821	7366	9899	47%	63%	34%	86%	0%
Number of pregnant women having 4 antenatal care (ANC) visits	15,821	2248	4866	14%	31%	116%	52%	1%
Number of women of reproductive age receiving family planning commodities	92,588	19,770	25,270	21%	27%	28%	22%	0%
Number of deliveries conducted by skilled staff	15,821	1464	2480	9%	16%	69%	27%	1%
Number of HIV positive mothers receiving prevention of mother to child transmission services	1252	409	469	33%	37%	15%	100%	0%
Number of long-lasting insecticide treated nets (LLITN) distributed to pregnant women	15,821	3162	8763	20%	55%	177%	100%	2%
Childhood								
New born with low birth weight^b^								
Number of new borne receiving BCG	15,821	12,844	17,182	81%	109%	34%	56%	1%
Number of children under one fully immunized	15,821	10,224	15,671	65%	99%	53%	86%	6%
Number of children under one vaccinated against measles	15,821	10,318	15,677	65%	99%	52%	86%	1%
Number of children receiving vitamin A	68,867	11,620	26,765	17%	39%	130%	76%	1%
Number underweight among children under five visiting health providers^c^		284	93			-67%	4%	0%
Number of children under five attending growth monitoring clinic	69,446	12,096	18,774	17%	27%	55%	80%	0%
Number of LLITN distributed to children under five years	69,446	3188	22,669	5%	33%	611%	87%	1%
Number of children under five treated for malaria	69,446	41,968	33,374	60%	48%	-20%	99%	3%
Number of children over five treated for malaria	316,337	126,630	89,742	40%	28%	-29%	71%	1%
Number of school children correctly de-wormed at least once a year	126,265	11,693	43,536	9%	34%	272%	98%	0%
Adolescence								
Number of health facilities offering the standard package of youth – friendly health services^d^								
Adulthood/all lifecycles (25–59 years)								
Number of HIV positive cases receiving anti-retroviral treatment	16,204	384	661	2%	4%	72%	13%	0%
Number of Voluntary counseling and testing clients	204,806	7044	12,299	3%	6%	75%	23%	0%
Number of tuberculosis (TB) cases detected^d^		98	124			27%		
No. of TB patients cured (sputum negative)^e^	98	21	97	21%	99%	362%		
No of TB patients who have completed treatment^e^	98	29	98	30%	100%	238%		
Community-level indicators								
Number of functioning community health workers	No baseline							
Number of households with access to clean water and latrine	No baseline							
Number of households sprayed with insecticide (IRS)	No baseline							
Number of trained village health committees^b^	No baseline							

a Difference (delta) in % coverage between baseline and target.

b So few babies are delivered in a facility that this indicator did not have a meaningful baseline. Furthermore, all but one facility set a target of zero. Therefore, percent changes were not meaningful.

c Eligible population unknown.

d No baseline and no targets set for this indicator.

e Note that the number of TB cases detected in the district was less than 100 and number treated was less than 30 at baseline therefore the proposed increase, although modest in absolute numbers, is large when calculated as a percent. Furthermore, these numbers were so small when stratified by facility that the maximum and minimums are not reported.

**Fig. 2:**
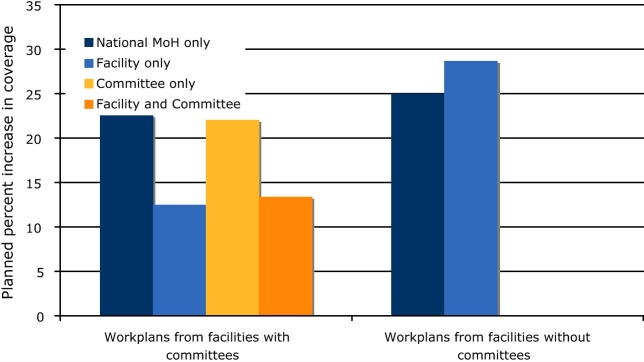
This graph shows the percent increase in planned coverage for key indicators grouped by which stakeholders in the planning process identified the indicator as a priority. Indicators were identified as priority by the National Ministry of Health only (i.e. not identified as a priority locally), the facility only, the community committee only, or both the facility and the committee. They are grouped by facilities that involved committees in the planning process and those which did not.

In order to understand how the committee input influenced the setting of targets, the following calculations were performed: the facility plan and committee minutes (where available) were reviewed for each facility to determine which participants in the planning process identified the indicator as a priority. Indicators were identified as priority by the (1) National Ministry of Health only (i.e. not identified as a priority locally), (2) the facility only, (3) the community committee only, or (4) both the facility and the committee. For each facility, the percent increase from baseline to target was calculated for each of these categories of indicators (1–4). For example, facility X identified childhood immunization, malaria, and ANC uptake as priority areas. The average increase in the targets relative to baseline for these three indicators was 30%. Facilities that did not convene their committee had only 2 groups of indicators–indicators that represented priorities identified by staff (category 2) and indicators that did not (category 1). The percent change in indicators was then compared between facilities that convened committees (*n* = 15) and those that did not (*n* = 7) (Fig. 2).

For facilities where committees provided input into the planning process, target increases were evenly distributed between facility priorities, community priorities, and mutual priorities. Targets were higher for facility priorities when no community feedback was available, indicating that target setting was influenced by community participation. Interestingly, for all facilities, targets were highest for indicators which reflected neither community nor facility reported priorities. For example, vitamin A supplementation was never mentioned as a priority intervention by facilities or communities, but very aggressive targets were set around this indicator. This reflected the responsiveness of the planning process to indicators chosen according to national priorities, even if they were not identified as local priorities.

There were several local priorities that were not represented by any indicator (Box 3), including filariasis, skin infections, bilharzias, diarrheal diseases, and chronic conditions such as hypertension, diabetes and arthritis. Communities listed dozens of issues affecting adults and the elderly, but all of the national indicators were focused on maternal and child health with the exception of indicators specific for HIV and TB.

Box 3 **Example of commonly-cited community priorities not represented in the national indicators (number of committees).**

**Table:** 

Childhood	Diarrhea (6) Skin diseases (4) Respiratory infections (4) Bilharzia (2) Jiggers (2) Snake bites (2)
Adolescence	Early pregnancy (5) Drug abuse (4)
Adult and elderly	Eye problems (4) Hypertension (3) Filariasis (3) Diabetes (3) Arthritis (3) Drug and alcohol abuse (3) Nutrition in the elderly (3)

Following target setting, activities were planned to achieve targets. Targets were only set for national indicators. Because targets were set first and activities were planned around those targets, no activities were planned or budgeted for that addressed local priorities *not* reflected by specific indicators.

## 7 Impact of facility level planning on district and facility budgets

As stated above, it was intended that the consolidated facility plans would feed into district and provincial level planning, and that funds would be released directly to facilities through the direct facility funding (DFF) to support their work plans. Although facility work plans were compiled directly into the district and provincial plans, the “resource envelope” dedicated to each facility, within which they budgeted, was never distributed. There was little evidence that financial allocations from the national level to each district for the year were influenced by the facility level planning activities. Moreover, the planned national roll out of DFF did not take place during the timeframe of AOP4 (it is currently planned during 2010–2011).

## 8 Discussion

In a study examining district-level planning undertaken nearly 10 years ago in Kenya, a major concern that emerged was the need to ‘close the gap between those who formulate policy and those who are expected to implement it’ [10]. More generally, health systems reform processes have increasingly recognized the essential contribution of communities to the success of health programs and development activities in general. The planning process in 2008 was designed to incorporate community priorities and community-designated activities through an interactive series of meetings with elders, community-owned resource persons, community members and facility staff. The first planning “unit” was intended to be the “community unit”. However, the absence of established community units and formalized government community-based health programs made this type of engagement impossible. Our experience shows that there is momentum at the national level to engage local stakeholders all the way down to the community, but significant gaps between policy makers and policy implementers still exist. These gaps still create challenges for those implementing policy.

In the absence of the community units, community involvement in the planning process was solicited through community members who had already been designated to represent their villages at the local government health facility. This representation may have been less comprehensive than envisioned, and input could not be generated by consensus within the broader community. However, the established working relationships between the facility staff and the committee certainly expedited the planning process and may have formed the basis for an effective working relationship that made a measurable impact on the outcome of the planning process. This is in contrast to studies in Tanzania [11], [12] that found little impact of community participation in planning and priority setting, despite planning guidelines and the presence of similar health committees at the facility and village levels.

Community priorities reflected an understanding of the broader context of health, and included education, environmental health, water and sanitation, in addition to functions traditionally performed by the health sector. The resulting work plans show that facilities weighed the priorities of the community when setting coverage targets. Facilities that convened community committees likely developed workplans that more closely reflected community priorities than those facilities that did not convene committees.

An unforeseen consequence of the planning guidelines was the impact of the national indicators on the outcome of the planning process. Target setting, activity planning and budgeting revolved around specific health service indicators that were chosen by the National Ministry of Health. While many of the communities’ priorities were represented by the indicators, others were not. The planning and budgeting process was not conducive to including these priorities in the annual workplan. Furthermore, facilities and districts are required to report on standardized national indicators to the national level and their performance is evaluated by measuring progress on these indicators, creating a disincentive for facilities to plan and budget for health priorities not represented by the indicators. The end result was a work plan that was more reflective of national priorities than local priorities. Similar observations have been made in Tanzania, where annual plans seemed to be largely dictated by national and donor priorities despite the emphasis on decentralization of decision-making and budgeting. The authors in Tanzania also found significant differences between the communities’ perceived needs and the actual district plans [12]. These challenges have been noted more widely in the decentralization and community accountability literature. For example Mills et al. [13] have noted that power is often not released by central government to intermediate and local levels, and that the process can in fact result in the complication of lines of responsibility and accountability. A comparative analysis of four countries undergoing decentralization of their health systems demonstrated little success in effectively engaging community participation, either through democratic representation in facility boards, local government, or village health committees [14]. It was noted that merely legislating roles for community participation at the facility or through committees was not effective. Further complexities in community participation include difficulties in defining ‘communities’ and their ‘representatives’, and differing perceptions and motivations among key stakeholders at national and local levels regarding if and how to involve communities [13], [15], [16], [17], [18], [19], [20], [21].

An important question that arises from the differing perspectives reported in the literature, and from the planning process we report in this paper, is what weight should be given to evidence-based planning versus demand-based planning. In other words, how accurately do the priorities identified by the community reflect the true burden of disease? For example, many committees listed filariasis as a priority problem in their communities. Is this because there is a high prevalence of this disease or because a person suffering from filariasis makes a strong visual impact and lasting impression? Is there a bias towards emphasizing health concerns that one's family has recently experienced? To what extent do cultural perceptions of health and disease influence prioritization of such issues? In other words, does the gap between the national indicators and the communities’ priorities reflect a difference in actual disease burden, a difference in the importance they attach to certain conditions, or a difference in their view of the scope of issues that should be addressed through the public health system? Should the health system strive to address the concerns of the community or focus on empirical evidence of disease burden and cost-effectiveness?

Several programs have used an epidemiological approach to priority setting in which local health officials undertake a situation analysis or act on locally derived data [22], [23]. However, emphasis on disease as an indicator of health service delivery problems may detract from understanding systems problems. The Kenya approach described here is a departure from the locally focused epidemiologic (or evidence-based) approach. The guidelines emphasized problem identification around barriers to health on the demand side and the supply side, rather than simply disease identification. Although this approach has the potential to identify service and systems problems rather than just disease priorities, it conflicted somewhat with the disease-specific focus of the indicators. It suggests there was a mismatch between the disease-specific indicators used for target setting and the barrier analysis in which the committees participated.

There are obvious limitations to this small, observational study. Although Kilifi is in many ways a typical district in Kenya, care should always be taken when extrapolating a single experience to a broader context. We are not aware of any other documentation of the implementation of the AOP4 guidelines. However, the experience in Kilifi is probably typical of the majority of districts in Kenya in many ways. Firstly, Kilifi operates with similar administrative structures and capacity at the district level. Secondly, few if any districts have been able to implement the government's community strategy comprehensively. In many districts, a small fraction of the population is served by community health workers within non-governmental programs, or alternatively a small number of government Community Units are sponsored by similar organizations. However, each district is likely to have had to adapt the planning process to account for the lack of CORPs.

However, Coast Province, which includes Kilifi district, was a pilot area for DANIDA funded health systems activities that included the direct facility funding (DFF) program [24]. The DFF program was the precursor to the facility-based financial management described above. Implementation of the DFF required each facility to have an active facility committee and to work with the committee to budget for a small amount of money available for basic operations expenses. As a result, facility committees were active in Kilifi and, together with the facility staff, had training and some experience in developing workplans and managing expenditures. Although every district in Kenya should have committees at each facility to represent the community, many of those committees are not active. It is not known how other districts executed planning at the facility level in the absence of community units or active facility committees. Thus the presence of active facility committees in Kilifi may have provided a better opportunity for the facility-based planning approach to work than in other districts that may have lacked both active committees and good coverage of CORPs.

A further limitation of this study was that data were not collected from the broader community, in order to assess how well they were represented by the facility staff and/or community individuals on the facility committees. The planning process occurred over a short period of time and it is unlikely that committee members were able to consult community members to solicit their input. As noted above, lack of confidence in representatives to accurately reflect community interests has been raised as a problem in other reports [25], [26].

In an oft-cited quote, Brownlea [27] states that ‘community participation is supposed to make a difference, but not simply to become a process’. Other studies have raised concerns about the responsiveness of the national level to priorities or initiative taken at the district and community levels [22], [26]. The ultimate success of Kenya's strategy for community engagement in health planning and priority setting will depend on how the information is used at the national level and whether the communities can see that their participation has made an impact, particularly on resource distribution. The work plans described in this report were never funded by the national level, although the ongoing DFF pilot project in Coast Province continued to provide a very small amount of money which may have allowed some of the activities planned by Kilifi district facilities to be implemented. Overall, this is a wasted investment in terms of money and man-hours applied to the planning process (including two weeks of intensive activities and overtime by the DHMT, transport to and from remote facilities, sitting fees for committees, and several days of closed clinics when in-charges were occupied by planning), but in terms of the communities’ investment. Community resources such as energy, participation, and engagement should not be viewed as unlimited. Unless some tangible return on their investment becomes apparent, community participation may not be as readily available in the future.

These reflections on the annual health planning process highlight some of the challenges of implementing national guidelines. There is a need for more prospective operational research aimed at informing policy guidelines in order to maximize the use of resources, including community input.

## 9 Conclusion

The experience in Kilifi highlights the feasibility and potential impact of engaging the community in the health planning process. The community representatives have a broad view of health issues and challenges in their communities. There is potential to incorporate views of the community through representatives at the facility level. The experience shows that even limited community involvement can influence health sector planning and may allow for activities and investments to be tailored to local needs, but this can easily be over-ridden by national frameworks for target setting. The challenge lies in ensuring that community views are responded to by the health system. More flexibility should be given to the districts and facilities to respond to community priorities, while observing a balance between evidence-based and demand-based planning.
